# Brain-Derived Neurotrophic Factor (BDNF) protein levels in anxiety disorders: systematic review and meta-regression analysis

**DOI:** 10.3389/fnint.2013.00055

**Published:** 2013-07-29

**Authors:** Sharain Suliman, Sian M. J. Hemmings, Soraya Seedat

**Affiliations:** ^1^MRC Anxiety Disorders Unit, Department of Psychiatry, Faculty of Medicine and Health Sciences, University of StellenboschCape Town, South Africa; ^2^Division of Molecular Biology and Human Genetics & Department of Psychiatry, Faculty of Medicine and Health Sciences, University of StellenboschCape Town, South Africa; ^3^Department of Psychiatry, Faculty of Medicine and Health Sciences, University of StellenboschCape Town, South Africa

**Keywords:** anxiety, BDNF, protein, meta-regression, systematic review

## Abstract

**Background:** Brain-Derived Neurotrophic Factor (BDNF) is a neurotrophin that is involved in the synaptic plasticity and survival of neurons. BDNF is believed to be involved in the pathogenesis of several neuropsychiatric disorders. As findings of BDNF levels in anxiety disorders have been inconsistent, we undertook to conduct a systematic review and meta-analysis of studies that assessed BDNF protein levels in these disorders.

**Methods:** We conducted the review using electronic databases and searched reference lists of relevant articles for any further studies. Studies that measured BDNF protein levels in any anxiety disorder and compared these to a control group were included. Effect sizes of the differences in BDNF levels between anxiety disorder and control groups were calculated.

**Results:** Eight studies with a total of 1179 participants were included. Initial findings suggested that BDNF levels were lower in individuals with any anxiety disorder compared to those without [Standard Mean Difference (SMD) = −0.94 (−1.75, −0.12), *p* ≤ 0.05]. This was, however, dependent on source of BDNF protein [plasma: SMD = −1.31 (−1.69, −0.92), *p* ≤ 0.01; serum: SMD = −1.06 (−2.27, 0.16), *p* ≥ 0.01] and type of anxiety disorder [PTSD: SMD = −0.05 (−1.66, 1.75), *p* ≥ 0.01; OCD: SMD = −2.33 (−4.21, −0.45), *p* ≤ 0.01].

**Conclusion:** Although BDNF levels appear to be reduced in individuals with an anxiety disorder, this is not consistent across the various anxiety disorders and may largely be explained by the significantly lowered BDNF levels found in OCD. Results further appear to be mediated by differences in sampling methods. Findings are, however, limited by the lack of research in this area, and given the potential for BDNF as a biomarker of anxiety disorders, it would be useful to clarify the relationship further.

## Background

Brain-Derived Neurotrophic Factor (BDNF) is a neurotrophin (NT) which promotes the proliferation, survival and differentiation of neurons in the peripheral and central nervous systems (Lindsay et al., 1994; Aydemir et al., 2006). Although BDNF is more concentrated in brain tissue, it is present in the bloodstream and derives from different sources, including platelets and brain (Yamamoto and Gurney, 1990; Radka et al., 1996; Lommatzsch et al., 2005). There have been reports that BDNF can cross the blood–brain barrier (Pan et al., 1998) and positive correlations between peripheral BDNF protein levels and brain levels have been reported in rodents (Karege et al., 2002a,b), suggesting that peripheral BDNF levels may reflect BDNF levels in the brain. BDNF blood levels have also been shown to correlate with cortical integrity (Lang et al., 2007). In clinical settings, peripheral blood levels (i.e., serum or plasma) are thus widely used as a proxy for central levels.

Although our understanding of BDNF expression remains incomplete, hormones such as estradiol and testosterone, as well as glucocorticoids (GCs), have emerged as important mediators of BDNF expression and function, and there have been suggestions of functional interaction between BDNF and GCs, such as in the regulation of corticotrophin-releasing hormone and other important neuropeptides (Carbone and Handa, 2013). BDNF may thus possibly regulate the response to stress through transmitter systems that regulate the hypothalamus-pituitary-adrenal (HPA) axis (Smith et al., 1995; Champagne and Meaney, 2001; Duval et al., 2004). Early life stress may continue to exert effects into adulthood by repeated activation of stress-responsive biological mediators such as GC and catecholamines (McEwen and Stellar, 1993; McEwen, 1998). Notably, BDNF and other neurotrophic factors are believed to counteract the negative impact of stress hormones on hippocampal volume (Duman, 2002; Manji et al., 2003). BDNF has been shown to be involved in anxiety-like behaviors in animal models, and numerous types of stressors have been found to cause reduced expression of BDNF (Hartmann et al., 2001; Duman, 2002; Rasmusson et al., 2002).

BDNF is also thought to be involved in the pathogenesis of several neuropsychiatric disorders, and numerous studies have examined BDNF protein levels in humans, mostly in relation to depression (Karege et al., 2005; Molendijk et al., 2011a). Although BDNF lacks diagnostic specificity, findings of BDNF alterations across a number of psychiatric disorders, underscores the shared common pathophysiological mechanisms and high rates of comorbidity (Sen et al., 2008). Serum BDNF levels have been shown to correlate with antidepressant efficacy and to predict an individual's response to antidepressant treatment at an early time point following treatment initiation. For example, recent meta-analyses have confirmed significantly lower BDNF protein levels for depressed patients relative to concentrations found in healthy controls (Bocchio-Chiavetto et al., 2010), with levels normalizing after treatment with antidepressants (Brunoni et al., 2008; Sen et al., 2008). Work has also shown that the combination of early serum BDNF non-increase plus early non-improvement on the Hamilton Depression Rating Scale predicted final treatment failure with 100% specificity (Tadić et al., 2011). These findings are mirrored by recent preliminary evidence of epigenetic (methylation) changes in the BDNF gene and inadequate antidepressant response in major depression (Tadić et al., 2013). These findings suggest that BDNF levels are likely to be a biomarker for depression and associated disorders, and support the notion that improvement of symptoms might be associated with the neuroplastic changes achieved by antidepressant treatment (Hashimoto, 2010). Furthermore, BDNF levels may help the clinician to predict clinical outcome. For example, findings by Kurita et al. (2012) indicate that if plasma BDNF levels decrease or are unchanged in an individual with regularly measured plasma BDNF, the clinician may need to re-evaluate treatment strategy.

Additionally, studies in human post-mortem brains have shown an involvement of BDNF in the pathophysiology of stress-related psychopathologies, such as mood and anxiety disorders (Duman and Monteggia, 2006; Carola et al., 2008; Dunham et al., 2009) and a review by Molendijk et al. (2012) suggests that BDNF expression contributes to psychopathological characteristics. Given the high level of comorbidity between depressive and anxiety disorders, and the similarities in their pathophysiology (Kendler et al., 1992, 1995; Klaassen et al., 1998; Maron et al., 2004; David et al., 2009), it is plausible that BDNF levels in anxiety disorders may mirror the changes found in depression and potentially serve as a peripheral biomarker. Findings of BDNF protein levels in anxiety disorders, in animal models (Chen et al., 2006; Govindarajan et al., 2006; Monteggia et al., 2007) and in humans (Maina et al., 2010; Molendijk et al., 2011b; Wang et al., 2011) have, however, been inconsistent. To date, there has been no published meta-analysis of BDNF protein levels in anxiety. Clarifying these findings is important in elucidating the relevance and clinical application of BDNF measures in the diagnosis and treatment of anxiety disorders. Further investigation of the predictive clinical utility in guiding antidepressant treatment in randomized controlled trials is warranted as it may have implications for the treatment of anxiety disorders and guide more rationale prescribing.

We thus undertook to systematically review all studies, in particular controlled studies, of BDNF protein levels [in serum, plasma, or cerebrospinal fluid (CSF)] in anxiety disorders [acute stress disorder (ASD), agoraphobia (AGP), generalized anxiety disorder (GAD), obsessive-compulsive disorder (OCD), phobia, panic disorder (PD), posttraumatic stress disorder (PTSD), social phobia/ social anxiety disorder (SAD)]. We sought, firstly, to assess whether BDNF protein levels were lower in individuals with anxiety disorders than in those without, and secondly whether there was any specificity of effect with regards to the different anxiety disorders (ASD, AGP, GAD, OCD, phobia, PD, PTSD, SAD).

## Methods

### Criteria for considering studies for this review

We included any study that assessed for BDNF protein levels in adults or children with an anxiety disorder compared to a control group, regardless of sample size or ethnic background. Diagnoses were determined by psychiatric interviews, based on DSM-IV or ICD-10 criteria, in all studies. The outcome of interest was plasma, serum or CSF BDNF expressed as picograms (pg) or nanograms (ng).

### Search strategy for identification of studies

Studies were identified through the following databases: Academic Search Premier, Africa Wide Information, CAB Abstracts, CINAHL, E-Journals, Eric, Healthsource: Nursing/Academic Edition, Pubmed and PsycArticles, between 18 June and 27 September 2012. Reference lists of pertinent articles were searched to identify any further relevant studies.

Search terms included a combination of “BDNF” or “brain-derived neurotrophic factor” with each of the following: “acute stress disorder,” “agoraphobia,” “anxiety,” “generalized anxiety disorder,” “obsessive-compulsive disorder,” “phobia,” “panic,” “posttraumatic stress disorder,” “social anxiety disorder,” “social phobia,” “stress.” No limit on the time period was applied to the search in order to avoid omission of relevant studies but the searches were restricted to the English language and humans. We excluded systematic and non-systematic review articles and studies of no direct relevance to the review.

Titles and abstracts of all original research articles identified were screened for eligibility and any abstract deemed potentially relevant was then reviewed in full text. The search and data-extraction were conducted separately by the first 2 authors and any uncertainty was discussed amongst the authors.

### Data extraction and analyses

Information extracted included study and population characteristics, sample size, study design, and outcomes relevant to this review. Means and standard deviations of BDNF levels were extracted and used to calculated the standard mean difference (SMD) and 95% confidence interval (CI) for individual studies and where appropriate to conduct meta-analyses. Information not available in a research article was obtained through the authors of that publication, where possible. We used the inverse-variance statistical method and, as we expected significant heterogeneity, a random-effects analyses model. Subgroup analyses were performed for the different anxiety disorders, if there was more than one study in the group. Sensitivity analyses were also performed to determine if results differed depending on whether (1) only studies assessing serum levels and (2) only studies assessing plasma levels were included.

## Results

**Figure 1:**
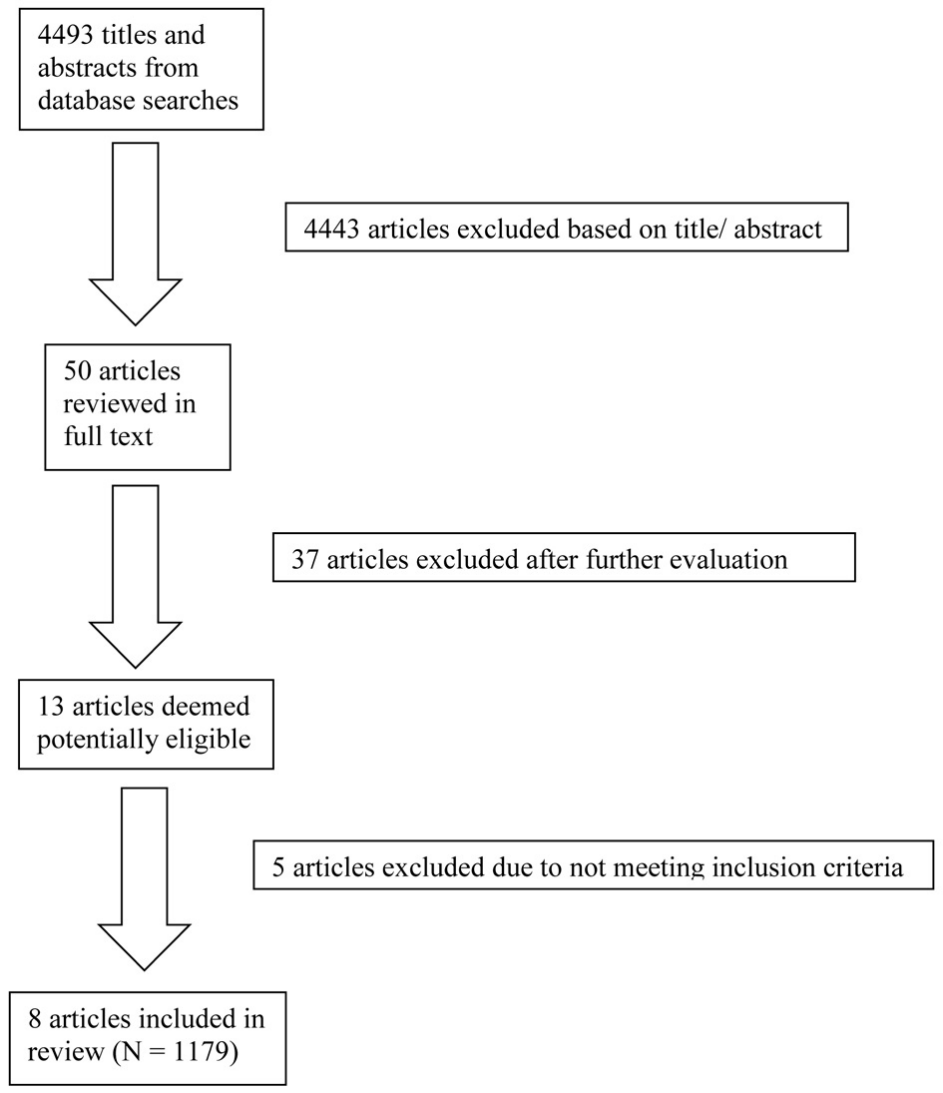
**Flow diagram of review process**.

The database searches yielded a total of 4493 results. Fifty abstracts were identified as potentially relevant and full manuscripts were obtained. Of these, 37 were excluded as they were not of relevance to the present review. Full texts of the 13 studies that appeared relevant were then reviewed in greater detail, and eight studies, with a total of 1179 participants, were found to meet inclusion criteria (see Figure 1). The following information was uniformly extracted from each study: first author's name, year of publication, source of publication, study characteristics (where study took place, study type), participant characteristics (age, gender, type of anxiety disorder, number of cases, and controls), measures of anxiety and BDNF. The reviewed studies are summarized in Tables 1 (table of included studies) and 2 (table of excluded studies). There was heterogeneity in sample characteristics, study methodology and measures of outcome i.e., BDNF protein levels were measured as serum, plasma and CSF and different units of measurement were used across studies (ng or pg/ml and pg/μg), limiting comparability. We report all BDNF levels in pg/ml (1 ng/ml = 1000 pg/ml or pg/μg).

**Table 1:** **Included studies**.

**Author**	***N* (Anxiety** ±)	**Setting and participants**	**Study type and design**	**Outcomes of interest and assessment measures**	**BDNF levels in patients vs. controls (mean, *SD, p*-value)**
Bonne et al., 2011	25 (16/11)	USA; 16 medication-free outpatients with chronic PTSD, 12 female; 11 non-traumatized healthy controls, 7 female; ages 18–65	Interventional study; cohort assessed pre- and post- treatment with paroxetine	PTSD; Diagnosis: SCID-CV (First et al., 1996) Severity: CAPS Blake et al., 1995 BDNF: CSF	1.00 ± 0.52 vs. 0.83 ± 0.44; *p* > 0.05 (pg/ml)
Dell'Osso et al., 2009	36 (18/18)	Italy; 18 medication-free outpatients with PTSD, 12 female; 18 non-traumatized healthy controls, 11 female); ages 18–65	Cross-sectional, case controlled study	PTSD; Diagnosis: SCID-I/P (First et al., 1995) Severity: IES (Horowitz et al., 1979) BDNF: plasma	5300 ± 1100 vs. 7400 ± 1500; *p* < 0.001 (pg/ml)
dos Santos et al., 2011	50 (25/25)	Brazil; 25 medication-free outpatients with OCD, 21 female; 25 healthy controls, 20 female; ages 18–60	Cross-sectional, case controlled study	OCD; Diagnosis: SCID-I/P (First et al., 1995) Severity: Y-BOCS (Goodman et al., 1989) BDNF: serum	0.470 ± 0.038 vs. 0.747 vs. 0.060; *p* < 0.001 (pg/μ*g*)
Hauck et al., 2010	68 (34/34)	Brazil; 34 outpatients with ASD or PTSD (21 recent trauma, 13 remote trauma), 27 female; 41% of patients using psychotropic medication; 34 age- and gender-matched healthy controls), 27 female; ages 14–65	Cross-sectional, case controlled study	PTSD; Diagnosis: MINI -Portuguese version (Sheehan et al., 1998; Amorin, 2000) Severity: DTS (Davidson et al., 1997) BDNF: serum	0.49 ± 0.21 vs. 0.25 ± 0.14, *p* < 0.01 (pg/μ*g*)
Maina et al., 2010	48 (24/24)	Italy; 24 medication-free outpatients with OCD and no recent psychological stressors, 9 female; 24 healthy age- and gender-matched controls, 9 female; ages 18–65	Cross-sectional, case controlled study	OCD; Diagnosis: SCID (First et al., 1996, 1997) Severity: Y-BOCS (Goodman et al., 1989) BDNF: serum	36,900 ± 6420 vs. 41,590 ± 7820; *p* < 0.05 (pg/ml)
Molendijk et al., 2011a	775 (393/382)	Netherlands; 393 medication-free outpatients with any anxiety disorder except OCD, 262 female; 382 healthy controls, 237 female; ages 18–65	Cross-sectional, cohort study	Anxiety Disorders: Diagnosis: CIDI (Wittchen et al., 1991) Severity: not assessed; BDNF: serum	9310 ± 3380 vs. 9490 ± 3180; *p* > 0.05 (pg/ml)
Strohle et al., 2010	24 (12/12)	Germany; 12 medication-free outpatients with PD (10 with Agora), 9 female; 12 age- and gender-matched controls, 9 female; ages 18–65	Interventional, case-controlled study; participants assessed pre- and post-excercise	PD; Diagnosis: MINI (Sheehan et al., 1998) Severity: the panic and agoraphobia scale (Bandelow, 1997) BDNF: serum	2700.2 ± 4184.9 vs. 9700.45 ± 7291.15; *p* < 0.01 (pg/ml)
Wang et al., 2011	137 (74/63)	China; 22 medication-free and 52 medication-treated outpatients with OCD, 26 female; 63 age and gender matched controls, 30 female; ages 18–64	Cross-sectional, case controlled study	OCD; Diagnosis: MINI (Sheehan et al., 1998) Severity: Y-BOCS (Kim et al., 1990) BDNF: plasma	1980 ± 1590 vs. 4090 ± 2000; *p* < 0.01 (pg/ml)

**Table 2:** **Excluded studies**.

**Author**	**Reason for exclusion**
Grassi-Oliveira et al., 2008	MDD patients with and without childhood physical neglect; PTSD assessed and severity of symptoms correlated with BDNF levels
Hauck et al., 2008	Case reports
Kauer-Sant'Anna et al., 2007	Bipolar patients with and without lifetime trauma exposure
Kobayashi et al., 2005	BDNF assessed post treatment only
Yoshimura et al., 2006	Case reports

### Description of studies

#### Included studies

Three studies assessed BDNF protein levels in patients with PTSD or ASD and compared them to non-traumatized, healthy controls. The Bonne study (Bonne et al., 2011) was conducted in the USA. It was an interventional study that assessed CSF BDNF levels in a cohort of 16 medication-free, non-combat related PTSD patients and 11 control participants. Participants with alcohol or substance abuse or dependence in the last six months were excluded; however, participants with current or past depression and other anxiety disorders were not excluded. In patients with PTSD, BDNF levels were assessed prior to and post 12 weeks of treatment with paroxetine. The PTSD group was older (mean age 36 ± 11.4 years) and comprised a greater proportion of females (75%) than the control group (mean age 35.3 ± 13.1 years, 64% female). Patients with PTSD were more likely than controls to have more anxiety and depressive symptoms and to meet criteria for past MDD. For the purpose of this review we only considered pre-treatment data. Patients with PTSD were found to have similar concentrations of BDNF when compared to healthy controls (1 ± 0.52 pg/ml vs. 0.83 ± 0.44 pg/ml, *p* > 0.05).

Dell'Osso et al. (2009) conducted a cross-sectional, case-controlled study in Italian participants. Plasma BDNF levels were assessed in 18 medication free outpatients with PTSD (67% female) and 18 healthy controls (61% female). Exclusion criteria included: current or lifetime diagnosis of organic mental disorder, schizophrenia, schizophreniform or other psychotic disorders, bipolar disorders, substance-related disorders, a current diagnosis of depressive disorder, uncontrolled or severe medical conditions, and any current or past psychopharmacological treatment. Patients had a mean age of 42.1 ± 12.5 years and controls of 38.8 ± 12.1 years. BDNF levels were significantly lower in patients than controls (5300 ± 1100 pg/ml vs. 7400 ± 1500 pg/ml, *p* < 0.001). BDNF levels did not correlate with any other demographic or clinical characteristic assessed.

The Hauck et al. (2010) study was also a cross-sectional, case-controlled study, conducted in Brazilian participants. Thirty-four outpatients with ASD or PTSD (21 who had experienced a traumatic event in the previous year, 13 who had experienced it more than 4 years before assessment) were compared with 34 age- and gender- matched healthy controls. Exclusion criteria comprised neurodegenerative disorders, psychotic symptoms, mental retardation, cancer and/or chronic/acute infection. Females comprised 79% of each group. More than a third (41%) of patients were using psychotropic medications at the time of assessment. Patients were younger (mean age 35.2 ± 13 years) than controls (mean age 36.2 ± 9.2 years) and had significantly higher serum BDNF levels than controls (0.49 ± 0.21 pg/μg vs. 0.25 ± 0.14 pg/μg, *p* < 0.001). However, when the patient group was stratified by recent and remote trauma, and compared with controls, only those who had experienced recent trauma had significantly higher BDNF levels (*p* < 0.001). The authors noted that the two patient groups did differ in terms of PTSD symptoms. However, there were no correlations between BDNF levels and any of the clinical rating scales used. A limitation of this study is that not all patients were drug-free, although this was not found to interfere with BDNF levels in the sample.

Three studies assessed BDNF protein levels in OCD patients and compared them to healthy controls. dos Santos and colleagues (2011) conducted a cross-sectional, case-controlled study comparing 25 un-medicated (medication free for >60 days) OCD outpatients with 25 healthy controls. Exclusion criteria considered were: current history of alcohol or any other substance use or abuse, history of encephalic/brain trauma followed by posttraumatic amnesia, current history of any other neurological or systemic disorders (i.e., epilepsy, Parkinson's disease, or systemic lupus), current use of medication that could lead to any psychopathological manifestations, current suicide risk, pregnant or breast-feeding women, <18 and >60 years of age, and a cognitive deficit that could result in an inability to understand the instruments and questionnaires. Mean age of patients was 44.1 ± 2.82 years and of controls was 37.44 ± 3.09 years. Eighty four percent of the OCD group was female compared to 80% in the control group. Body mass index (BMI) was significantly lower in the OCD group, but BDNF levels were not found to correlate with BMI. Sixty four percent of patients had comorbid depression and 44% a comorbid anxiety disorder (PTSD: 24%; PD: 24%; SAD: 20%; GAD: 16%; AGP: 8%). In addition, 8% had skin-picking disorder and 4% body dysmorphic disorder. Serum BDNF levels were significantly lower in the OCD group compared to controls (0.0470 ± 0.038 pg/μg vs. 0.747 ± 0.060 pg/μg: *p* < 0.001). Within the patient group, sexual/ religious symptom content, chronic course of symptoms and the presence of depression or SAD resulted in elevated BDNF levels compared to those without these features.

Maina et al. (2010) assessed serum BDNF levels in 24 medication-free OCD outpatients with no recent psychological stressors, and in 24 age- and gender-matched healthy controls. The following exclusion criteria were considered: current or previous diagnosis of organic mental disorder, schizophrenia, or other psychotic disorders, bipolar disorders, substance-related disorders; current diagnosis of depressive disorder and a maximum total score of 7 on the Hamilton Depression Rating Scale 17-item (HAM-D-17); uncontrolled or serious medical condition; any current or past psychopharmacological treatment; any severe stressful event within the year prior to inclusion. The mean ages of patients and controls were 37.7 ± 12.2 years and 38.2 ± 10.6 years, respectively, and 38% of each group was female. The case-control study was conducted in Italy and was cross-sectional in design. BDNF levels were significantly decreased in the OCD group compared to controls (36900 ± 6420 pg/ml vs. 41590 ± 7820 pg/ml; *p* = 0.043). BDNF levels were not correlated with any clinical characteristic assessed, but there was a trend for lower BDNF levels in patients with a history of major depression (*p* = 0.09).

Wang et al. (2011) assessed plasma BDNF in 22 medication free (27% female) and 52 drug-treated (36% female) outpatients with OCD and 63 age- and gender-matched controls (48% female), in China. This, too, was a cross-sectional, case-controlled study. Individuals were excluded if they met any other DSM-IV axis I diagnosis, including lifetime history of depression; had a Hamilton Depression Rating Scale (HAMD, 17-item) score >7 (Hamilton, 1960); had any prior or current suicide attempts; were pregnant or lactating; or were in physical health such that they could not complete the study. BDNF concentrations differed significantly between patients and controls with both medication free (1970 ± 1800 pg/ml, *p* = 0.00) and drug treated (1980 ± 1540 pg/ml, *p* = 0.00) OCD patients having lower levels than controls (4090 ± 2000 pg/ml). There was no significant difference in BDNF concentrations between medication-free and drug-treated patients, but females in the medication-free group had lower BDNF levels than males in the same group (*p* = 0.04). OCD patients tended to be younger than controls and length of illness in drug treated patients was higher than in medication-free patients. For the purposes of this review, we combined the medication-free and drug treated OCD groups to get a combined BDNF mean of 1980 ± 1590 pg/ml.

One interventional study assessed BDNF levels in patients with Panic Disorder and one in a combined group of anxiety disorder patients. Strohle et al. (2010) studied 12 medication-free outpatients with PD (10 with agoraphobia) and 12 age- and gender- matched controls in the German population. Any participants with a comorbid Axis I disorder and using antidepressants were excluded from the study. Seventy-five percent of each group was female. Patients were found to have low depression and moderate anxiety levels. Serum BDNF levels were assessed prior to and post 30 min of exercise or quiet rest. Patients with panic disorder had significantly reduced serum BDNF levels compared to controls before rest or exercise (*p* = 0.003). For the purpose of this review we used the baseline assessments provided to us by the authors and combined these to get a combined BDNF mean of 2700.2 ± 4184.9 pg/ml for patients and 9700.45 ± 7291.15 pg/ml for controls.

Molendijk et al. (2011a) reported on a cross sectional analysis of a longitudinal study, in which serum BDNF levels were assessed in a cohort of 393 medication-free anxiety disorder patients and 382 healthy controls. Exclusion criteria included comorbid MDD and psychotropic medication use. The study took place in the Netherlands and participants were recruited from mental health care services, primary health care services and the general population. Sixty-seven percent of patients and 62% of controls were female. The patient group comprised participants with Agoraphobia, GAD, PD, SAD, or a combination of these disorders. All were currently free of current depression but 45% has a history of past depression. Controls were free of lifetime mood and anxiety disorders and not at high risk for these disorders. On average, patients were younger, had fewer years of education, and were more likely to smoke and/ or use alcohol, but these variables were controlled for in the analyses. BDNF concentrations did not differ significantly between patients and controls (9310 ± 3380 pg/ml vs. 9490 ± 3180 pg/ml, *p* = 0.49). There were also no statistically significant differences in BDNF levels between each type of anxiety disorder vs. controls. However, female patients had lower BDNF levels than female controls and male patients.

#### Excluded studies

Five studies were excluded from the review. Two studies were excluded as they were case reports on serum BDNF levels (Yoshimura et al., 2006; Hauck et al., 2008) and one only assessed serum BDNF levels after medication treatment (Kobayashi et al., 2005). Another study assessed serum BDNF levels in patients with bipolar disorder and compared those with and without trauma exposure (Kauer-Sant'Anna et al., 2007). Although the authors report that participants with and without PTSD did not differ with regards to BDNF levels, not enough information was available to enable us to compare the two groups. The final study compared patients with major depressive disorder with and without childhood physical neglect (Grassi-Oliveira et al., 2008). Although PTSD was assessed for, comparisons with regards to plasma BDNF were not reported, and again not enough information was available for us to compare the groups.

### Association of BDNF with anxiety disorders

The forest plot of included studies is presented in Table 3. Six of the eight studies included showed differences in BDNF protein levels between participants with an anxiety disorder and participants without. In five of these reports (Dell'Osso et al., 2009; Maina et al., 2010; Strohle et al., 2010; dos Santos et al., 2011; Wang et al., 2011), BDNF levels (both serum and plasma) in patients with an anxiety disorder were lower than in participants without an anxiety disorder, with effect sizes ranging from moderate to large. The finding of one report (Hauck et al., 2010) was, however, in the opposite direction, with patients having significantly higher BDNF levels than controls. Effect size in this study was large. In the final 2 studies (Bonne et al., 2011; Molendijk et al., 2011a) participants with an anxiety disorder did not differ from those without with regards to BDNF levels. When results of all studies were combined, there was a significant difference between groups with lower BDNF levels in patients with an anxiety disorder [SMD = −0.94 (−1.75, −0.12); *z* = 2.25, *p* = 0.02]. There was, however, also significant statistical heterogeneity across studies (*p* < 0.01; *I*^2^ = 95%).

**Table 3:** **Meta-regression analysis of BDNF in anxiety disorders**.

**Study or subgroup**	**Anxiety**			**Control**			**Weight %**	**Std. mean difference IV, random, 95% CI**	**Std. mean difference IV, random, 95% CI**
	**Mean**	**SD**	**Total**	**Mean**	**SD**	**Total**			
Bonne et al., 2011	1	0.52	16	0.83	0.44	11	12.3	0.34 [−0.44, 1.11]	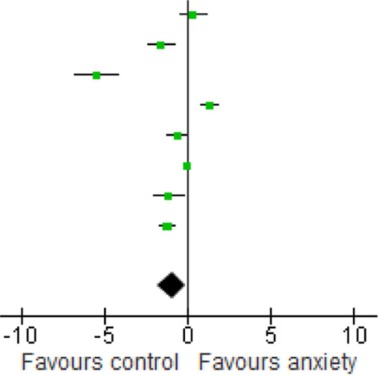
Dell'Osso et al., 2009	5300	1100	18	7400	1500	18	12.3	−1.56 [−2.32, −0.80]	
dos Santos et al., 2011	0.47	0.038	25	0.75	0.06	25	10.4	−5.49 [−6.74, −4.24]	
Hauck et al., 2010	0.49	0.21	34	0.25	0.14	34	13.1	1.33 [0.80, 1.86]	
Maina et al., 2010	36,900	6420	24	41,590	7820	24	12.9	−0.64 [−1.23, −0.06]	
Molendijk et al., 2011a	9310	3380	393	9490	3180	382	13.8	−0.05 [−0.20, 0.09]	
Strohle et al., 2010	2700.2	4184.9	12	9700.45	7291.15	12	11.9	−1.14 [−2.01, −0.26]	
Wang et al., 2011	1980	1590	74	4090	2000	32	13.3	−1.22 [−1.66, −0.77]	
Total (95% Cl)			596			538	100.0	−0.94[−1.75, −0.12]	

Heterogeneity: τ^2^ = 1.26; χ^2^ = 145.79, df = 7 (P < 0.00001); 1^2^ = 95%.

Test for overall effect: Z = 2.25 (P = 0.02).

Sensitivity analyses of serum studies (Table 4) yielded a different result, although there was a trend toward significance for lower BDNF levels in the anxiety disorders [SMD = −1.06 (−2.27, 0.16); *z* = 1.70, *p* = 0.09]. Sensitivity analyses of plasma studies alone did, however, result in significant group differences [SMD = −1.31 (−1.69, −0.92); *z* = 1.70, *p* < 0.01], with little heterogeneity present (*p* > 0.05; *I*^2^ = 0) (Table 5).

**Table 4:** **Sensitivity analysis: studies using serum**.

**Study or subgroup**	**Anxiety**			**Control**			**Weight %**	**Std. mean difference IV, random, 95% CI**	**Std. mean difference IV, random, 95% CI**
	**Mean**	**SD**	**Total**	**Mean**	**SD**	**Total**			
Bonne et al., 2011	1	0.52	16	0.83	0.44	11	0.0	0.34 [−0.44, 1.11]	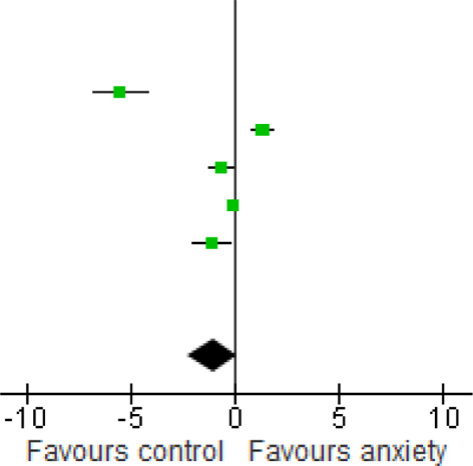
Dell'Osso et al., 2009	5300	1100	18	7400	1500	18	0.0	−1.56 [−2.32, −0.80]	
dos Santos et al., 2011	0.47	0.038	25	0.75	0.06	25	17.6	−5.49 [−6.74, −4.24]	
Hauck et al., 2010	0.49	0.21	34	0.25	0.14	34	20.8	1.33 [0.80, 1.86]	
Maina et al., 2010	36,900	6420	24	41,590	7820	24	20.6	−0.64 [−1.23, −0.06]	
Molendijk et al., 2011a	9,310	3380	393	9490	3180	382	21.6	−0.05 [−0.20, 0.09]	
Strohle et al., 2010	2700.2	4184.9	12	9700.45	7291.15	12	19.4	−1.14 [−2.01, −0.26]	
Wang et al., 2011	1980	1590	74	4090	2000	32	0.0	−1.22 [−1.66, −0.77]	
Total (95% Cl)			488			477	100.0	−1.06 [−2.27, 0.16]	

Heterogeneity: τ^2^ = 1.77; χ^2^ = 108.59, df = (P < 0.00001); 1^2^ = 96%.

Test for overall effect: Z = 1.70 (P = 0.09).

**Table 5:** **Sensitivity analysis: studies using plasma**.

**Study or subgroup**	**Anxiety**			**Control**			**Weight %**	**Std. mean difference IV, random, 95% CI**	**Std. mean difference IV, random, 95% CI**
	**Mean**	**SD**	**Total**	**Mean**	**SD**	**Total**			
Bonne et al., 2011	1	0.52	16	0.83	0.44	11	0.0	0.34 [−0.44, 1.11]	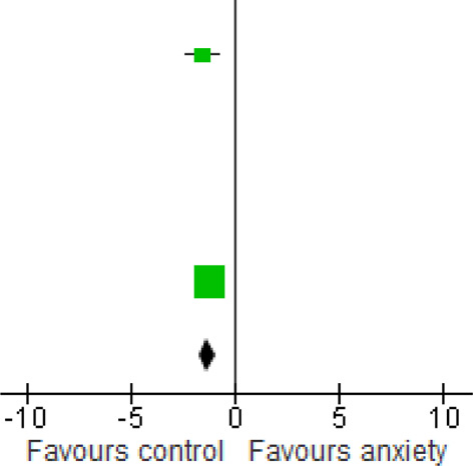
Dell'Osso et al., 2009	5300	1100	18	7400	1500	18	25.9	−1.56 [−2.32, −0.80]	
dos Santos et al., 2011	0.47	0.038	25	0.75	0.06	25	0.0	−5.49 [−6.74, −4.24]	
Hauck et al., 2010	0.49	0.21	34	0.25	0.14	34	0.0	1.33 [0.80, 1.86]	
Maina et al., 2010	36,900	6420	24	41,590	7820	24	0.0	−0.64 [−1.23, −0.06]	
Molendijk et al., 2011a	9310	3380	393	9490	3180	382	0.0	−0.05 [−0.20, 0.09]	
Strohle et al., 2010	2700.2	4184.9	12	9700.45	7291.15	12	0.0	−1.14 [−2.01, −0.26]	
Wang et al., 2011	1980	1590	74	4090	2000	32	74.1	−1.22 [−1.66, −0.77]	
Total (95% Cl)			92			50	100.0	−1.31 [−1.69, −0.92]	

Heterogeneity: τ^2^ = 0.00; χ^2^ = 0.59, df = 1 (P = 0.44); 1^2^ = 0%.

Test for overall effect: Z = 6.65 (P < 0.00001).

When we looked at the PTSD studies only, we found no significant effect [SMD = −0.05 (−1.66, 1.75); *z* = 0.06, *p* = 0.95] (Table 6). Sub-analyses of OCD studies, however, did show a significant effect [SMD = −2.33 (−4.21, −0.45); *z* = 2.43, *p* = 0.02] (Table 7). Heterogeneity was high in both these analyses. Taking into account the significant effect in studies of OCD, we re-ran the analyses, excluding studies of OCD and found no significant effect [SMD = 0.18 (−1.00, 0.64); *z* = 0.42, *p* = 0.67] (Table 8).

**Table 6:** **Subgroup analysis: PTSD studies**.

**Study or subgroup**	**Anxiety**			**Control**			**Weight %**	**Std. mean difference IV, random, 95% CI**	**Std. mean difference IV, random, 95% CI**
	**Mean**	**SD**	**Total**	**Mean**	**SD**	**Total**			
Bonne et al., 2011	1	0.52	16	0.83	0.44	11	32.9	0.34 [−0.44, 1.11]	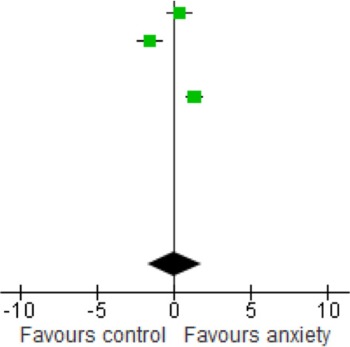
Dell'Osso et al., 2009	5300	1100	18	7400	1500	18	33.0	−1.56 [−2.32, −0.80]	
dos Santos et al., 2011	0.47	0.038	25	0.75	0.06	25	0.0	−5.49 [−6.74, −4.24]	
Hauck et al., 2010	0.49	0.21	34	0.25	0.14	34	34.1	1.33 [0.80, 1.86]	
Maina et al., 2010	36,900	6420	24	41,590	7820	24	0.0	−0.64 [−1.23, −0.06]	
Molendijk et al., 2011a	9310	3380	393	9490	3180	382	0.0	−0.05 [−0.20, 0.09]	
Strohle et al., 2010	2700.2	4184.9	12	9700.45	7291.15	12	0.0	−1.14 [−2.01, −0.26]	
Wang et al., 2011	1980	1590	74	4090	2000	32	0.0	−1.22 [−1.66, −0.77]	
Total (95% Cl)			68			63	100.0	0.05 [1.66, 1.75]	

Heterogeneity: τ^2^ = 2.15; χ^2^ = 37.69, df = 2 (P < 0.00001); 1^2^ = 95%.

Test for overall effect: Z = 0.06 (P = 0.95).

**Table 7:** **Subgroup analysis: OCD studies**.

**Study or subgroup**	**Anxiety**			**Control**			**Weight %**	**Std. mean difference IV, random, 95% CI**	**Std. mean difference IV, random, 95% CI**
	**Mean**	**SD**	**Total**	**Mean**	**SD**	**Total**			
Bonne et al., 2011	1	0.52	16	0.83	0.44	11	0.0	0.34 [−0.44, 1.11]	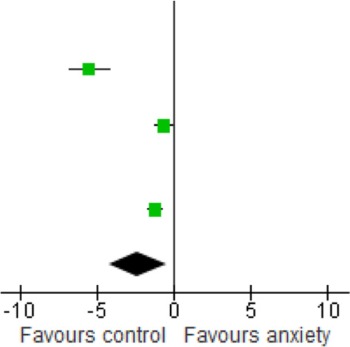
Dell'Osso et al., 2009	5300	1100	18	7400	1500	18	0.0	−1.56 [−2.32, −0.80]	
dos Santos et al., 2011	0.47	0.038	25	0.75	0.06	25	30.7	−5.49 [−6.74, −4.24]	
Hauck et al., 2010	0.49	0.21	34	0.25	0.14	34	0.0	1.33 [0.80, 1.86]	
Maina et al., 2010	36,900	6420	24	41,590	7820	24	34.4	−0.64 [−1.23, −0.06]	
Molendijk et al., 2011a	9310	3380	393	9490	3180	382	0.0	−0.05 [−0.20, 0.09]	
Strohle et al., 2010	2700.2	4184.9	12	9700.45	7291.15	12	0.0	−1.14 [−2.01, −0.26]	
Wang et al., 2011	1980	1590	74	4090	2000	32	34.9	−1.22 [−1.66, −0.77]	
Total (95% Cl)			123			81	100.0	−2.33 [4.21, −045]	

Heterogeneity: τ^2^ = 2.59; χ^2^ = 48.06, df = 2 (P < 0.00001); 1^2^ = 96%.

Test for overall effect: Z = 2.43 (P = 0.02).

**Table 8:** **Meta-regression analysis with OCD studies excluded**.

**Study or subgroup**	**Anxiety**			**Control**			**Weight %**	**Std. mean difference IV, random, 95% CI**	**Std. mean difference IV, random, 95% CI**
	**Mean**	**SD**	**Total**	**Mean**	**SD**	**Total**			
Bonne et al., 2011	1	0.52	16	0.83	0.44	11	19.0	0.34 [−0.44, 1.11]	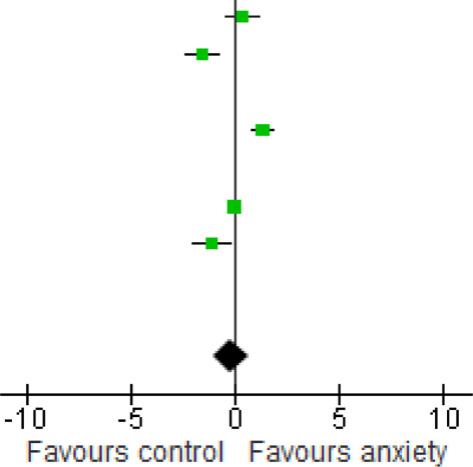
Dell'Osso et al., 2009	5300	1100	18	7400	1500	18	19.2	−1.56 [−2.32, −0.80]	
dos Santos et al., 2011	0.47	0.038	25	0.75	0.06	25	0.0	−5.49 [−6.74, −4.24]	
Hauck et al., 2010	0.49	0.21	34	0.25	0.14	34	20.9	1.33 [0.80, 1.86]	
Maina et al., 2010	36,900	6420	24	41,590	7820	24	0.0	−0.64 [−1.23, −0.06]	
Molendijk et al., 2011a	9310	3380	393	9490	3180	382	22.7	−0.05 [−0.20, 0.09]	
Strohle et al., 2010	2700.2	4184.9	12	9700.45	7291.15	12	18.2	−1.14 [−2.01, −0.26]	
Wang et al., 2011	1980	1590	74	4090	2000	32	0.0	−1.22 [−1.66, −0.77]	
Total (95% Cl)			473			457	100.0	0.05 [1.66, 1.75]	

Heterogeneity: τ^2^ = 0.76; χ^2^ = 48.31, df = 4 (P < 0.00001); 1^2^ = 92%.

Test for overall effect: Z = 0.42 (P = 0.67).

## Discussion

We conducted a comprehensive search to identify studies related to our research question and believe that our review gives the most comprehensive overview of BDNF in anxiety disorders to date. We used systematic methods to reduce bias in the identification of studies, data extraction and synthesis, and appraisal of study quality. Our initial results suggested that BDNF levels differ between individuals with any anxiety disorder compared to those without, with levels being lower in those with an anxiety disorder. This was, however, dependent on the source of BDNF protein. Studies using plasma samples found a significant effect, whereas studies in serum showed only a trend toward significance.

The discrepancies between plasma and serum findings may be attributable to differences in the constitution of plasma and serum, which consequently represent separate pools of physiological material (D'Sa et al., 2012). Indeed, a study in depressed patients suggests that plasma BDNF may be state-dependent marker while serum BDNF may be a trait marker; in the aforementioned study antidepressants normalized plasma BDNF levels only, in line with clinical improvement, and serum BDNF levels remain unchanged (Piccinni et al., 2008). Peripheral BDNF is largely stored in platelets and is released from activated platelets to serum during the clotting process, explaining the lower concentration of BDNF in plasma compared to serum (Rosenfeld et al., 1995; Radka et al., 1996). Further, differences in platelet functioning, either by their ability to release BDNF or sequester BDNF from blood, may result in differences between serum and plasma BDNF levels (Bus et al., 2011). There has been much debate over whether plasma or serum levels are an appropriate proxy for brain BDNF concentrations, with some authors noting that results for serum BDNF levels cannot be generalized to studies of BDNF in plasma or platelets (Bus et al., 2011).

Owing to the paucity of eligible studies, subgroup analyses of the different anxiety disorders were limited to PTSD and OCD studies. The PTSD subgroup analysis did not reveal any group differences in BDNF levels, however, the OCD analyses did find significantly reduced BDNF levels in patients with OCD compared to controls. While only plasma studies in PTSD found differences in the general direction of the meta-analysis, two serum studies found these differences in OCD. Although the dearth of eligible studies did not permit further analyses to ascertain whether differences are attributable to protein source, this does suggest a stronger biological effect of BDNF in OCD. Of note, when studies of OCD were excluded from the meta-analysis, group differences were no longer significant for the other anxiety disorders. This effect, therefore, seems to be restricted to OCD.

To date, the majority of studies examining BDNF in patients with anxiety disorders have focused on genetic associations between the *Val66Met* polymorphism and the development of anxiety, and have reported conflicting results (Alonso et al., 2008; Hemmings et al., 2008; Wendland et al., 2008). Results of this review and meta-analyses, focusing on BDNF protein levels from serum, plasma and CSF, indicate that lowered levels of BDNF in participants with anxiety disorders in comparison to controls is due to significantly lowered levels of BDNF in patients with OCD. The variation in the direction of association, or lack of association, between individual studies may represent differences in BDNF sampling and analysis (whether serum, plasma or CSF BDNF levels were measured, storage conditions, type of assays used, etc.), or underlying genetic differences between populations. Age, BMI, gender and sex hormones, smoking, alcohol use, physical activity, time of day of blood draw, and number of months of storage have also been found to play a role in BDNF levels in both healthy adults and in those with a psychiatric diagnosis (Lommatzsch et al., 2005; Trajkovska et al., 2007; Ziegenhorn et al., 2007; Li et al., 2009; Bus et al., 2011; Maina et al., 2010; Ozan et al., 2010; Molendijk et al., 2011a).

It is important to bear in mind that these findings are largely based on the assumption that peripheral BDNF levels mirror the amount of BDNF in the brain, and this may be a limitation since there are other potential sources of BDNF (Karege et al., 2002b). The brain may be a major contributor to circulating blood levels (Rasmussen et al., 2009), and BDNF has been found to cross the blood-brain barrier (Pan et al., 1998). It is therefore conceivable that peripheral BDNF levels may comprise BDNF that has originated in CNS neurons (Lommatzsch et al., 2005). To this end, a number of recent animal studies have documented a correlation between levels of BDNF in blood and brain (Karege et al., 2002a; Sartorius et al., 2009; Klein et al., 2011), although not all results have been consistent (Elfving et al., 2010; Lanz et al., 2012)

BDNF is synthesized as a precursor protein, known as proBDNF, which is subsequently cleaved to yield mature BDNF. Although it was initially thought that proBDNF was inactive, it has recently been found to play a role in a number of physiological functions, by binding to the pan-neurotrophin receptor p75 (Pang et al., 2004). In fact, the yin-yang neurotrophic theory posits that proBDNF and mature BDNF exhibit opposing effects on neuroplasticity, with proBDNF initiating apoptotic signaling cascades, whilst mature BDNF initiates cell survival pathways by binding to the tyrosine kinase B receptor (TrkB) (Lu et al., 2005). It may thus be beneficial to investigate the levels of proBDNF and mature BDNF separately; however, studies included in the present review utilized ELISAs which were not able to discriminate between the two forms of BDNF.

A number of other limitations deserve mention. First, since anxiety disorders and depression are highly comorbid and there is evidence for lower BDNF in depression, the inclusion of patients with depression in some of the studies may partially account for the reduced plasma BDNF levels in the patient sample. This, however, does not appear to be the case here as four of the five included studies (Hauck et al., 2010; Maina et al., 2010; Bonne et al., 2011; Wang et al., 2011) found that the presence of past and/ or current depression did not have a significant effect, while the fifth study found BDNF levels were in fact elevated in patients with MDD (dos Santos et al., 2011).

Second, two studies contained in the meta-regression included patients on psychotropic medication treatment (Hauck et al., 2010; Wang et al., 2011) and previous studies that have examined the relationship of BDNF and psychotropic medication use, (i.e., serotonin reuptake inhibitors), have shown that the latter medications increase peripheral BDNF levels in humans (Piccinni et al., 2008; Matrisciano et al., 2009). However, it should be noted that in both studies medication did not appear to have a significant effect on outcomes. Third, although the quality of included studies was judged overall to be adequate, the findings in the present study need to be interpreted with caution, given that not all studies reported on potential confounding variables and their adjustment in analyses. Further, meta-analytic data also need to be cautiously interpreted, given the substantial heterogeneity between studies.

In summary, although there is evidence for lower peripheral BDNF in anxiety disorders, this seems to result specifically from lower BDNF in OCD, rather than other anxiety disorders. However, given the paucity of studies and the relatively small number of participants in most, as well as the limitations noted above, the role of BDNF protein in anxiety disorders is still currently far from being understood. To this end, further evidence from large prospective studies is needed. In particular, studies that address the generalizability of serum and plasma samples, and the variable procedures that lead up to the measurement of peripheral BDNF are called for.

### Conflict of interest statement

The authors declare that the research was conducted in the absence of any commercial or financial relationships that could be construed as a potential conflict of interest.
